# Habitat management as a safe and effective approach for improving yield and quality of tea (*Camellia sinensis*) leaves

**DOI:** 10.1038/s41598-018-36591-x

**Published:** 2019-01-23

**Authors:** Jianlong Li, Ying Zhou, Bo Zhou, Hao Tang, Yiyong Chen, Xiaoyan Qiao, Jinchi Tang

**Affiliations:** 1 Tea Research Institute, Guangdong Academy of Agricultural Sciences and Guangdong Provincial Key Laboratory of Tea Plant Resources Innovation and Utilization, Dafeng Road 6, Tianhe District, Guangzhou, 510640 China; 2 Guangdong Provincial Key Laboratory of Applied Botany & Key Laboratory of South China Agricultural Plant Molecular Analysis and Genetic Improvement, South China Botanical Garden, Chinese Academy of Sciences, Xingke Road 723, Tianhe District, Guangzhou, 510650 China; 3 University of Chinese Academy of Sciences, No.19A Yuquan Road, Beijing, 100049 China

## Abstract

Tea (*Camellia sinensis*) leaves are used to make the most widely consumed beverage globally after water. Therefore, the safety and quality of raw tea leaves are important indices for making tea and related products. Habitat management has been widely used as an environmentally friendly method to control pests in agroecosystems. To investigate the impact of habitat management on tea plantation ecosystems, a habitat management approach with intercropping was established. The function of habitat management on pest control was evaluated. Furthermore, metabolome and transcriptome analysis were applied to assay changes in quality-related metabolites. The habitat management approach was found to maintain arthropod biodiversity and develop natural arthropod enemies in the tea plantation. Therefore, the yield of the habitat management-treated tea plantation was increased. Metabolome analysis showed that epigallocatechin-3-gallate, the major catechin in tea leaves, has a significantly increased content in leaves of tea plants under habitat management compared with those in the control tea plantation. The content of L-theanine, the major amino acid in tea leaves, was not significantly changed in tea plants under habitat management. Furthermore, aroma compounds were more abundant in tea leaves from the habitat management-treated tea plantation than those from the chemical pesticide-treated tea plantation. Therefore, habitat management is reported for the first time as a safe and effective approach to improving the yield and quality of tea leaves.

## Introduction

Tea (*Camellia sinensis* (L.) O. Kuntze) is an important commercial crop consumed worldwide, primarily as a beverage made from processed leaves. Tea pest control is the major challenge in tea plantation management. Globally, 1,034 arthropod species occur in tea plantations^1^. Approximately 3% of these arthropods are common pests, such as *Ectropis obliqua*, *Empoasca onukii*, and *Euproctis pseudoconspersa*, throughout the major tea-producing regions^1^. Outbreaks of these pests account for serious yield losses and quality reduction. Various pest control methods, such as chemical pesticides, sex pheromones, and entomopathogenic insect pesticides, have been applied globally^1^. Among these, the use of chemical pesticides is the predominant method used by farmers to control tea pests. However, excessive pesticide use to prevent and control pests pollutes the environment, resulting in ecological damage and reduced biodiversity. Pesticide residues also negatively affect food safety and result in health risks^2^. Other environmentally friendly pest control technologies, such as biological pesticides and sex pheromones, have some application limitations^3^. Therefore, new green, recyclable, and low-carbon-emission methods for the prevention and control of tea plantation pests are required.

In recent years, habitat management strategies have been applied and shown to be effective at controlling pests^4–7^. Habitat management involves intervening in the vegetation of an agroecosystem with the intention to suppress pest densities^5^. The rational distribution of crops makes it possible to increase the biological diversity of agroecosystems in time and space^8,9^, which facilitates the control of crop pests by the agroecosystem itself. Habitat management has been successfully applied to field pest control in rice, which is the major food crop of Asia^10^.

Habitat management and improved biodiversity can change the environment in an agroecosystem^5^. Plant metabolites can be altered through environmental adaption. Aroma compounds, amino acids, and polyphenols are the key taste and flavour-related metabolites that influence tea quality^11,12^. These metabolites can be changed by insect attacks^13,14^, shading treatment^15^, light quality^16^, and temperature^17–19^. Tea plants are perennial evergreen shrubs that can easily form a more stable and functional ecosystem. Therefore, habitat management may effectively control pests in tea plantations. However, the effect of habitat management on tea quality remains unknown. Our study aimed to evaluate the effect of habitat management on tea pest control and tea leaf quality. In addition to providing pest control, habitat management was proven to be a safe and effective method for improving tea quality.

## Results

### Effect of habitat management on tea plantation yield

Tea yield is the key factor impacting the economic value of a tea plantation. To evaluate the function of habitat management in tea plantations, the yields of the control (CK) and habitat management (HM) tea plantations were analysed from 2014 to 2016. Tea yield indices, including the lengths of one bud with two leaves, bud densities, and hundred-bud weights, were also analyzed. Table 1 shows that the yields of the HM and CK tea plantations were not significantly different in both 2014 and 2015. However, the yield of the HM tea plantation was significantly higher than that of the CK tea plantation in 2016. Furthermore, the chemical pesticide (CP)-treated tea plantation was also evaluated in 2016. Although the yields between the CP and HM tea plantations were not significantly different, the HM plantation had a significantly higher single bud with two leaves length and hundred-bud weight (Table 1).

**Table 1:** Tea yields of different tea plantations.

Year	Tea types	Length of one bud and two leaves (cm)	Density of bud (number/m^2^)	Weight of 100 buds (g)	Tea Yield (g/m^2^)
2014	CK	11.07 ± 0.16a	201.73 ± 5.52a	99.73 ± 1.75a	851.55 ± 27.08a
	HM	10.28 ± 0.26b	222.68 ± 7.95a	102.83 ± 2.69a	914.02 ± 44.20a
2015	CK	10.58 ± 0.29ab	226.51 ± 2.02a	105.61 ± 1.44b	953.44 ± 17.00a
	HM	11.15 ± 0.57a	216.56 ± 11.43a	114.43 ± 1.35a	992.29 ± 57.60a
2016	CK	8.64 ± 0.08a	205.85 ± 7.30a	90.79 ± 1.21a	735.84 ± 30.32b
	HM	8.43 ± 0.08a	247.17 ± 10.30b	86.73 ± 0.32b	867.78 ± 44.65a
	CP	7.46 ± 0.10b	299.20 ± 7.65c	77.21 ± 1.54c	914.57 ± 25.32a

Data are expressed as mean ± S.D. (n = 10). Different letters indicate significant differences (p < 0.05).

### Effect of habitat management on pest control and ecosystem stability

To evaluate the function of habitat management in pest control on tea plantations, the types and numbers of arthropods in the CK, HM, and CP tea plantations were recorded twice a month in 2016. The types of arthropods, such as natural enemies, pests, and neutral insects, in the three tea plantations were almost the same, belonging to 20 orders, 48 families, and 53 species.

The greatest number of natural enemies was found in the HM tea plantation. Araneida, including Salticidae and Linyphiidae, were the major natural enemies in the agroecosystem^20^. Table 2 shows that the number of Araneida individuals was significantly greater in the HM tea plantation, at 1.71-fold more than in the CK tea plantation and 3.83-fold more than in the CP tea plantation. CP applications reduced the number of natural enemies significantly. The number of natural enemies on the CP tea plantation was half that on the CK tea plantation. Neutral insects were also the most abundant on the HM tea plantation (Table 2).

**Table 2:** Analysis on the composition of arthropod community in different tea plantations in 2016.

Arthropods	CK	HM	CP
Araneida	54.8 ± 4.9b	111.3 ± 9.8a	26.7 ± 2.7c
Acarina	8.9 ± 2.0a	15.3 ± 3.1a	1.1 ± 0.4b
Mantodea	0.0 ± 0.0a	0.0 ± 0.0a	0.0 ± 0.0a
Hemiptera	5.4 ± 0.6b	7.9 ± 1.2a	2.8 ± 0.7b
Coleoptera	1.7 ± 0.6b	3.4 ± 0.7a	0.5 ± 0.1b
Diptera	14.8 ± 1.8a	18.8 ± 2.2a	7.2 ± 0.9b
Hymenoptera	37.8 ± 3.8b	80.3 ± 22.2a	16.7 ± 3.9b
Odonata	0.8 ± 0.3a	0.4 ± 0.2a	0.0 ± 0.0a
Dermaptera	0.8 ± 0.2a	1.2 ± 0.8a	0.1 ± 0.1a
**Natural enemies total**	125.1 ± 8.3b	238.4 ± 29.1a	55.0 ± 6.0c
Hemiptera	658.1 ± 77.9a	542.4 ± 61.0a	208.0 ± 15.2b
Lepidoptera	16.5 ± 2.4a	12.5 ± 3.0b	4.9 ± 1.4b
Orthoptera	2.8 ± 0.6a	2.6 ± 0.6a	3.3 ± 0.6b
Coleoptera	16.0 ± 3.2a	17.2 ± 2.3a	8.8 ± 1.1b
Diptera	0.0 ± 0.0a	0.2 ± 0.1a	0.0 ± 0.0a
**Pests total**	693.9 ± 79.1a	574.8 ± 62.5a	225.0 ± 16.2b
Isopoda	2.3 ± 0.5b	24.3 ± 4.3a	0.9 ± 0.4b
Polydesmida	0.8 ± 0.5a	0.3 ± 0.1a	1.9 ± 1.0a
Orthoptera	3.2 ± 0.6a	3.3 ± 0.6a	0.3 ± 0.1b
Isoptera	17.6 ± 5.4ab	39.5 ± 14.1a	1.7 ± 1.2b
Blattodea	9.3 ± 1.1b	33.2 ± 4.0a	4.1 ± 0.7b
Lepidoptera	5.4 ± 0.8a	8.1 ± 1.4a	5.4 ± 0.6a
**Neutral insects total**	38.6 ± 5.0	108.5 ± 19.8	14.3 ± 2.3
**Neutral insects: Natural enemies: Pests**	1: 3.2 : 18	1: 2.2: 5.3	1: 3.8: 15.7

Data are expressed as mean ± S.D. (n = 24). Different letters indicate significant differences (p < 0.05).

The major pests on the tea plantations were members of orders Hemiptera, Lepidoptera, and Coleoptera. Compared with the CK tea plantation, the greater number of natural enemies and neutral insects on the HM tea plantation significantly reduced the number of pests (Table 2). The ratio of natural enemies to pests was highest on the HM tea plantation.

The natural enemy-following phenomenon of spiders and small green leafhoppers, the most serious pest in the tea plantations, was observed in both the CK and HM tea plantations (Supplementary Figure S1). This natural enemy-following phenomenon was observed more clearly in the HM tea plantation (Supplementary Figure S1), but was not observed in the CP tea plantation. The arthropod diversity and richness indices of the plantations were in the order HM > CK > CP (Supplementary Table S1). Furthermore, the CP tea plantation had the highest dominance index (Supplementary Table S1). Among the three tea plantations, the diversity and richness levels of arthropods showed less variation in a time series in the HM tea plantation (Supplementary Figure S2), and the community was stable. The CP tea plantation showed a large fluctuation in a time series (Supplementary Figure S2).

### Effect of habitat management on tea product safety

To evaluate tea product safety, tea leaves from the three tea plantations were analysed for six types of pesticide residues. Three types of pesticides, chlorfenapyr, imidacloprid, and avermectin, were detected in the CP tea plantation, while none of the pesticide residues were detected in the CK and HM tea plantations (Table 3).

**Table 3:** Contents of pesticide residues in the three types of tea (mg/kg).

Variety of pesticide	CK	SJ	HX
Chlorfenapyr	n.d.	n.d.	n.d.—0.025
Imidacloprid	n.d.	n.d.	n.d.—0.027
Avermectins	n.d.	n.d.	n.d.—0.025
Beta cypermethrin	n.d.	n.d.	n.d.
Bifenthrin	n.d.	n.d.	n.d.
Isocarbophos	n.d.	n.d.	n.d.

Data are expressed as mean ± S.D. (n = 3).

### Metabolomes and transcriptomes in the three tea plantations

**Figure 1:**
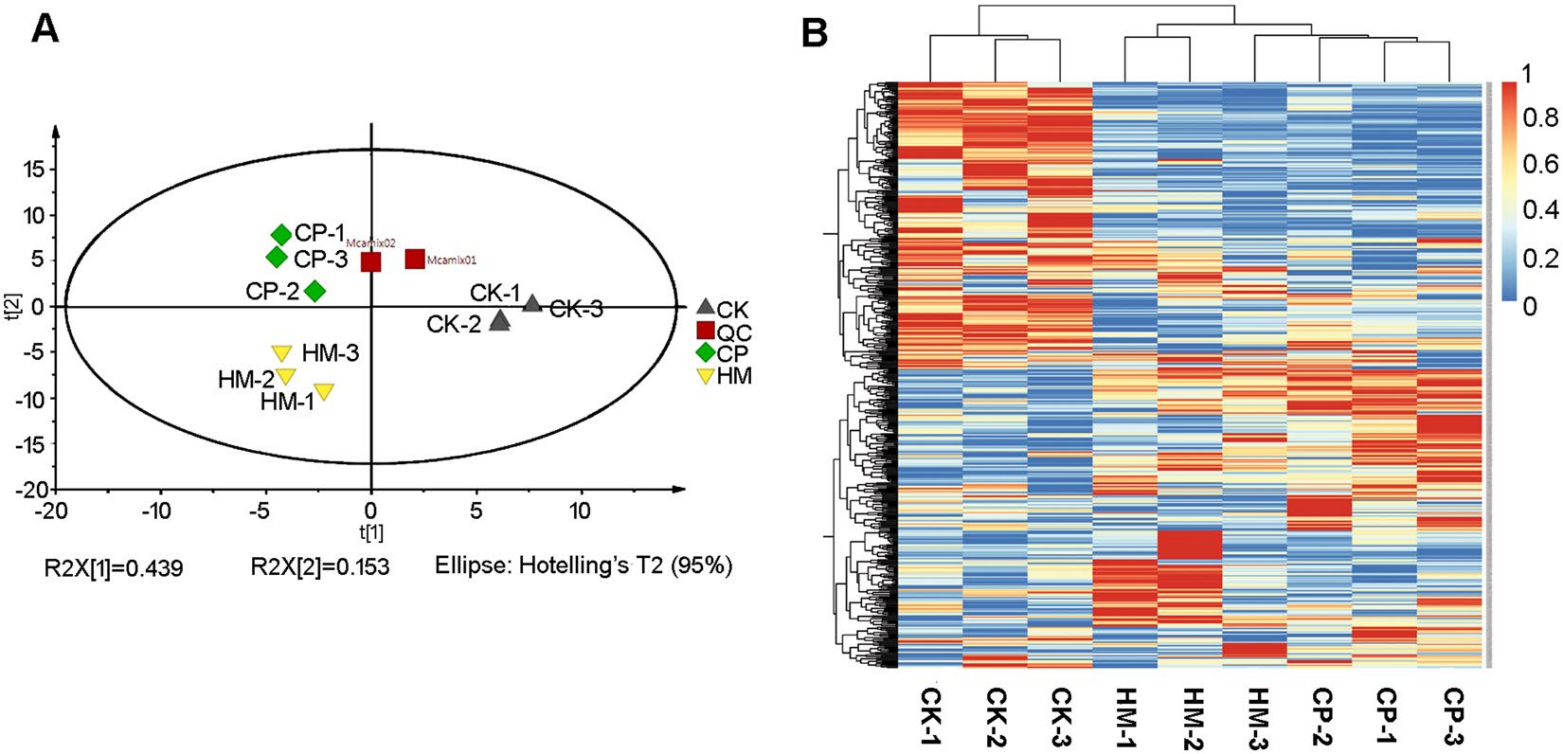
Comparison of metabolomic profiles among tea leaves from CK, HM and CP tea plantations. (**A**) PCA of metabolomes of tea leaves from CK, HM and CP tea plantations. (**B**) Heatmap of the different metabolites in tea leaves from CK, HM and CP tea plantations. CK, control; HM, habitat management; CP, chemical pesticide.

To further evaluate the effects of habitat management on tea quality, the metabolites in tea leaves grown on the three plantations were analysed using UPLC–MS/MS. A total of 511 metabolites were detected in the tea leaves, of which 79 were unknown metabolites. To determine the differences in metabolites produced among these treatments, principal component analysis (PCA) was performed. Figure 1A shows the results from the three plantations displayed in two dimensions. The PCA score plot showed that the metabolites in leaves from the three plantations were clearly differentiated, with the three biological replicates from each plantation clustering together. This suggested that the metabolic profiles from each plantation were significantly different and highly reproducible. These differences were larger between the CK and HM plantations and between the CK and CP plantations. The difference between the HM and CP plantations was relatively small. Figure 1B shows a heatmap of different metabolite data (three replicates for each plantation), which is in agreement with the PCA results. Many metabolites were present at their highest levels in tea plants from the CK plantation.

**Figure 2:**
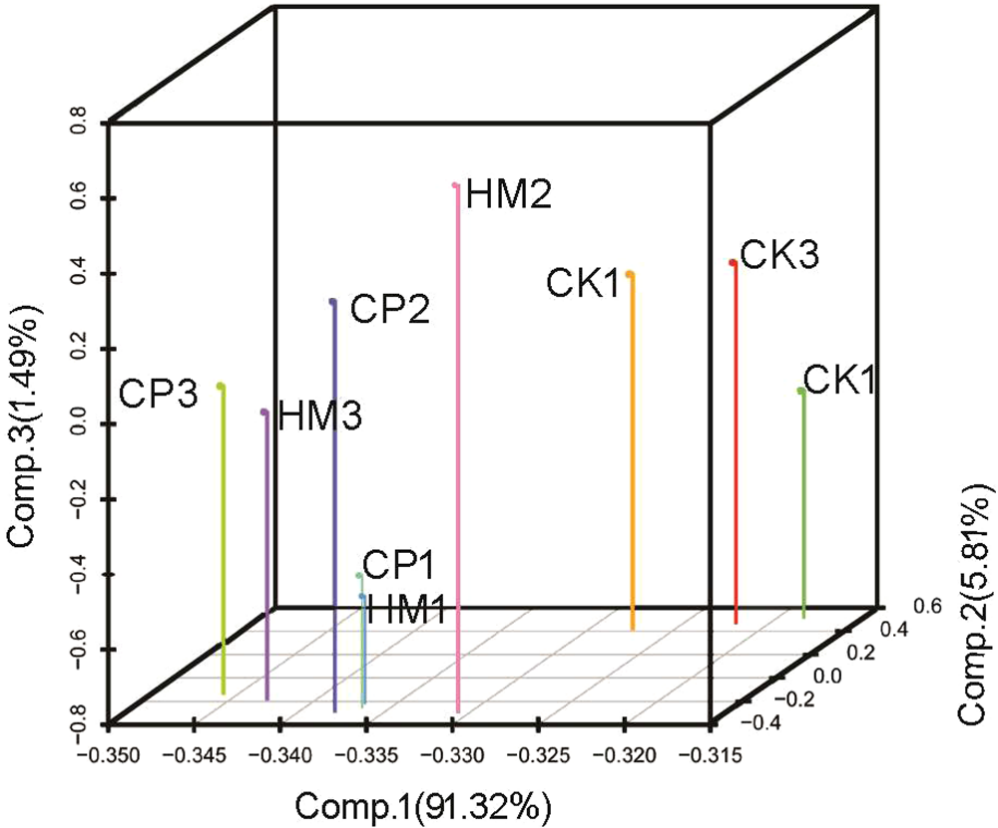
PCA of transcriptomes of tea leaves from CK, HM and CP tea plantations. CK, control; HM, habitat management; CP, chemical pesticide.

In accordance with the metabolome data, PCA of the transcriptome data showed that the CK and HM plantations were well differentiated, as were the CK and CP plantations (Fig. 2). The gene expression profiles of the HM and CP plantations were not significantly different according to PCA (Fig. 2).

### Effect of habitat management on contents of quality-related metabolites in tea leaves

**Figure 3:**
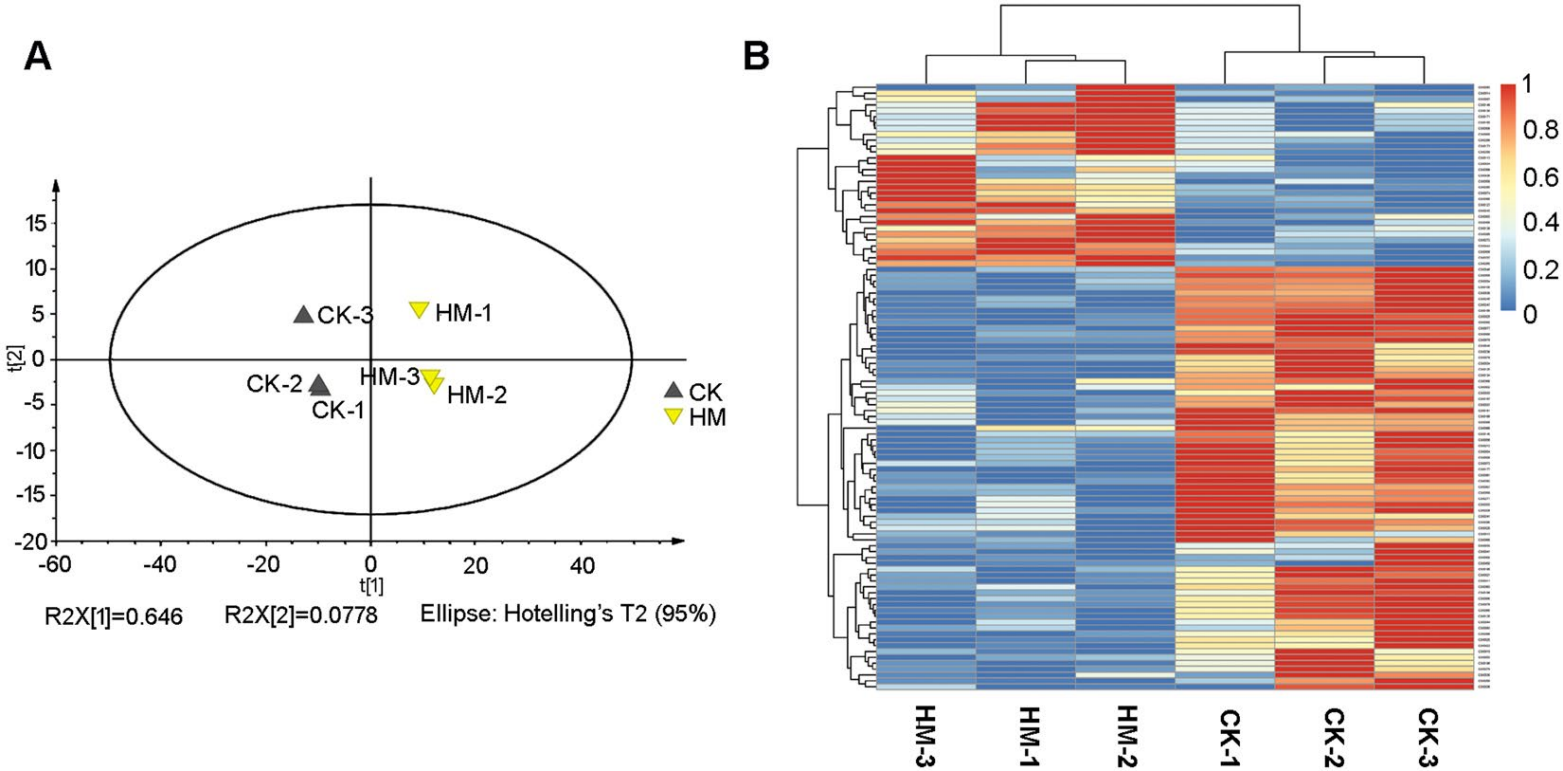
Comparison of metabolomic profiles between tea leaves from CK and HM tea plantations. (**A**) PCA of metabolomes of tea leaves from CK and HM tea plantations. (**B**) Heatmap of the different metabolites in tea leaves from CK and HM tea plantations. CK, control; HM, habitat management.

**Figure 4:**
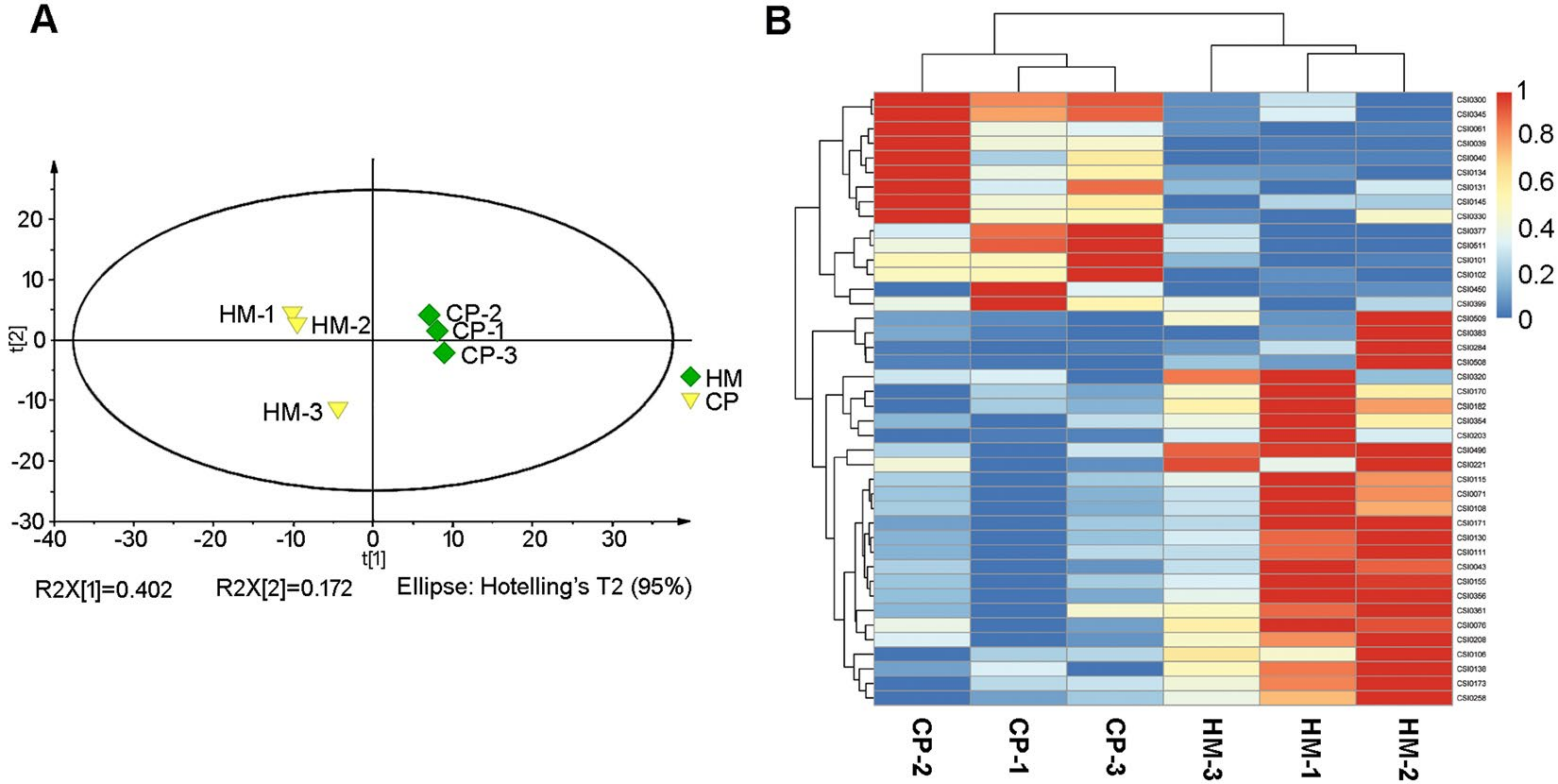
Comparison of metabolomic profiles between tea leaves from HM and CP tea plantations. (**A**) PCA of metabolomes of tea leaves from HM and CP tea plantations. (**B**) Heatmap of the different metabolites in tea leaves from HM and CP tea plantations. HM, habitat management; CP, chemical pesticide.

PCA and heatmap analysis suggested that the differences in metabolic profiles between the HM and CK plantations (Fig. 3A and B) and between the HM and CP plantations (Fig. 4A and B) were significant. In total, 103 differential metabolites were found in the HM and CK plantations. Phenylpropanoids, nucleotides and their derivatives, and catechin derivatives had significantly higher contents in leaves from the HM plantation. In total, 42 differential metabolites were found between the HM and CP plantations. Some nucleotides and their derivatives, organic acids and their derivatives, and tryptamines and their derivatives were present at higher levels in leaves from the HM plantation.

**Figure 5:**
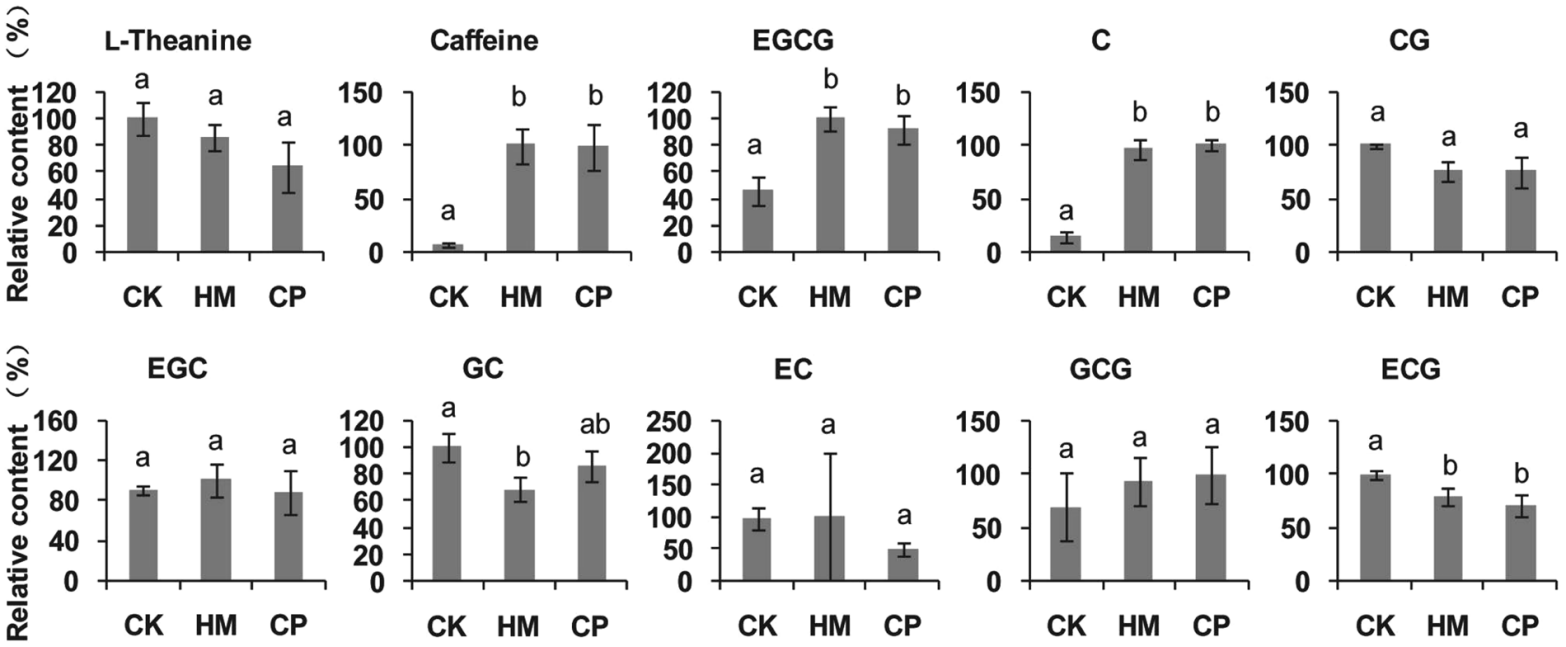
Relative contents of tea taste- and quality-related metabolites in tea leaves from CK, HM and CP tea plantations. The metabolites were analyzed by LC–MS/MS. The greatest content of each metabolite was set as 100%. EGCG, epigallocatechin-3-gallate; C, catechin; CG, catechin-3-gallate; EGC, eigallocatechin; GC, gallocatechin; EC, epicatechin; GCG, gallocatechin-3-gallate; and ECG, epicatechin-3-gallate. CK, control; HM, habitat management; CP, chemical pesticide.

Detailed metabolome analysis suggested that quality-related metabolism was altered by the habitat management (Fig. 5). Compared with the CK tea plantation, the caffeine content was significantly increased in tea leaves from the HM tea plantation. Epigallocatechin-3-gallate (EGCG), the most important catechin in tea leaves, showed a significantly increased content in tea leaves from the HM tea plantation, as did Catechin C. Other catechins, including catechin-3-gallate, epigallocatechin, epicatechin, and gallocatechin-3-gallate, did not show significantly different amounts between tea leaves from the CK and HM tea plantations. The L-theanine content was not significantly different between leaves from the CK and HM plantations. Compared with the CP tea plantation, the caffeine, L-theanine, and catechin contents in tea leaves from the HM tea plantation were not significantly different.

**Figure 6:**
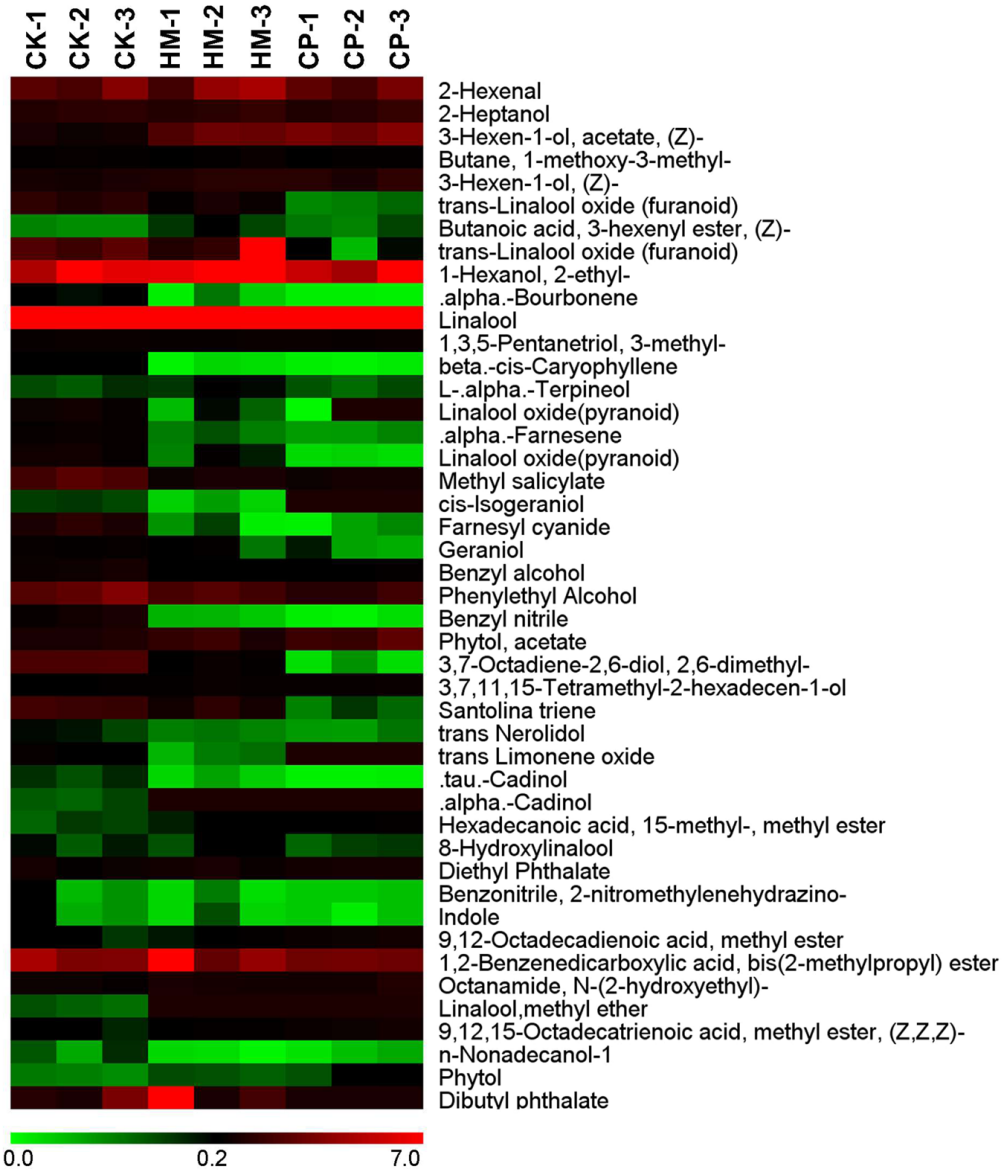
Heatmap of the relative aroma compoundcontentsof tea leaves from CK, HM and CP tea plantations. CK, control; HM, habitat management; CP, chemical pesticide.

**Figure 7:**
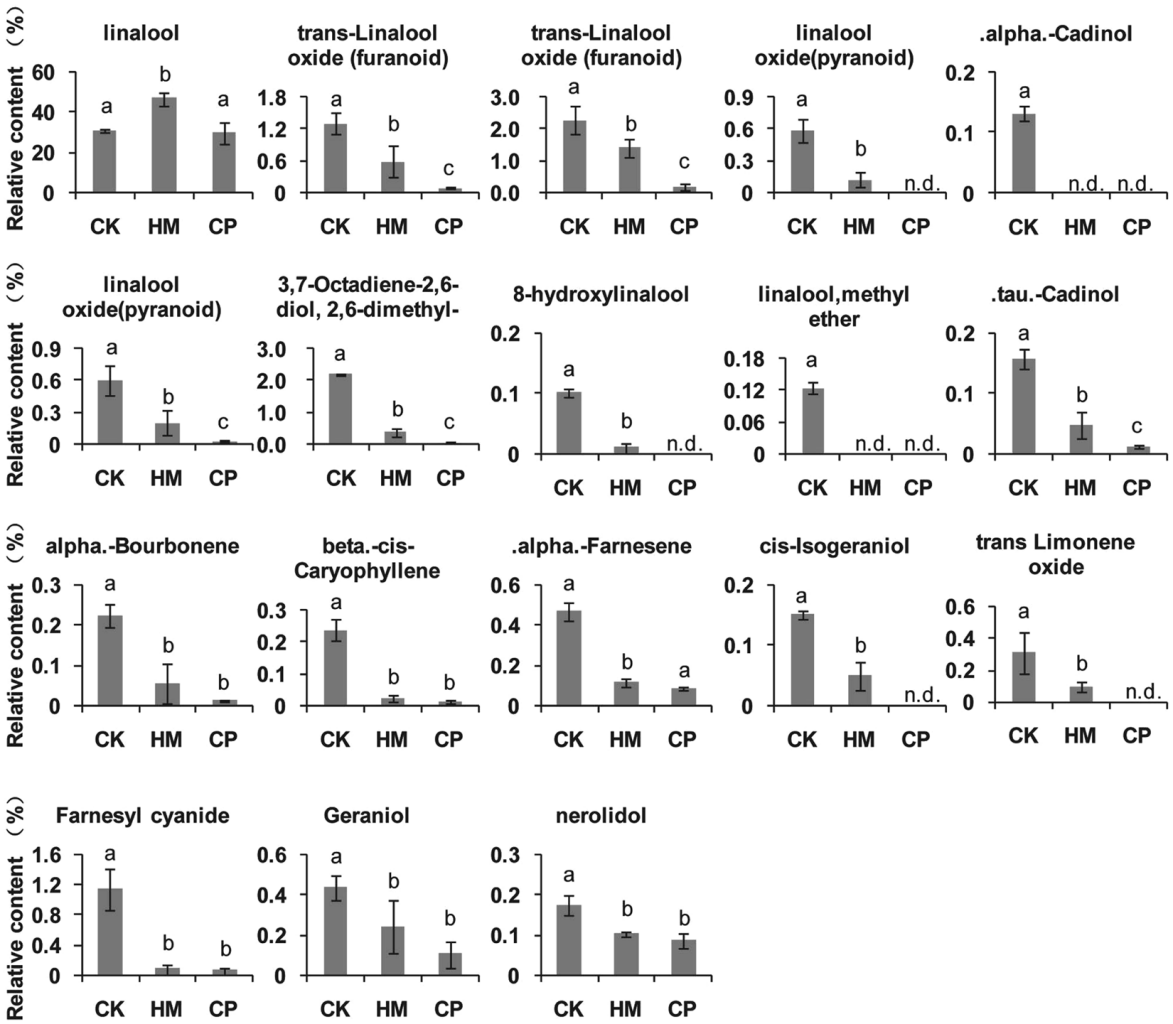
Relative terpenoid contents of tea leaves from CK, HM and CP tea plantations. The *y*-axis unit is the peak area ratio of the analyte to the internal standard. CK, control; HM, habitat management; CP, chemical pesticide.

Aroma is another key quality-related component. To analyse the aroma compound profiles of tea plants from the different plantations, the endogenous volatiles were extracted with CH_2_Cl_2_ and subjected to GC–MS analysis. The major endogenous volatile from Yinghong No.9 was linalool, comprising 41% of the total detected aroma compounds. Although most of the aroma compounds were present at their highest levels in tea leaves from the CK plantation, the linalool content was highest in tea leaves from the HM plantation (Figs 6 and 7). Compared with tea leaves from the CP tea plantation, aroma compounds, especially terpenoids, had significantly higher contents in tea leaves from the CK and HM tea plantations (Figs 6 and 7). Some aroma compounds were not even detected in tea leaves from the CP plantation.

## Discussion

Habitat management has been applied broadly to many agricultural systems^17,21,22^. In the present study, habitat management was found to be effective at maintaining tea plantation yields. Intercropping on the HM tea plantation might be responsible for the improved yield. Shading, which was provided by intercropping on the tea plantation, is beneficial to tea plant growth and improves tea plantation yields^23^. In addition to macrophanerophytes, hedgerow intercropping, such as with *Eupatorium*, also increases the yield by fertilizing the soil^24^.

The improved yield of the HM tea plantation might also be attributed to effective pest control resulting from the increases in natural enemy and neutral insect populations. The complex landscape was expected to enhance natural pest control^25^. As the landscape of the HM tea plantation was more suitable for natural enemy population growth, these natural enemies could effectively suppress pest populations. Neutral insects are also important in agroecosystems, providing food for natural enemies during crop early growth stages and benefitting natural enemy development^26,27^. In nature, the abundances of predatory natural enemies and neutral insects are positively correlated^28^.

Increased biodiversity is expected to improve ecosystem stability^29^. In the present study, habitat management was effective in forming a healthy ecosystem and providing ecological balance on the tea plantation. The fluctuating levels of arthropod diversity and richness after spring tea picking and during winter on the CP tea plantation suggested that this ecosystem was delicate. CP usage disrupted the balance between pests and natural enemies, which might have caused the resurgence in pests induced by pesticide resistance.

Another critical problem of CP application is pesticide residue. CP residue is a critical factor that impacts the safety of tea products. The use of CPs for pest control left pesticide residues on tea products. Presently, increased attention is being paid to food safety. Tea product safety was negatively affected by frequent CP use. Owing to health risks, strict management of CPs and residue monitoring have been implemented in most countries. Although CP application prevents yield losses caused by pests in the short-term, their residues are problematic to tea safety long-term. The results of this study suggest that habitat management can effectively replace CP usage to maintain tea plantation yields.

Yield and safety are just two of the indices used to evaluate the function of habitat management. Another factor closely related to the economic value of tea is tea quality. Metabolites are the foundation of tea quality, providing the basis for evaluating the nutritional and economic values of tea. Our study showed that different planting management strategies mediated gene expression and changed the metabolic profiles. Some tea quality indicators were improved under HM treatment.

L-Theanine and catechins are the major taste and health-related components in tea, and are the major components of the index used to evaluating tea functions^30^. L-Theanine gives tea its distinctive umami taste^31^ and can also induce a relaxed state, lower blood pressure, and improve learning abilities in humans^32^. The functions of catechins in vascular homeostasis, atherogenesis reduction, and cardiovascular disease prevention have also been well documented^33^. EGCG is the major catechin in tea leaves^34^. Higher EGCG and normal L-theanine contents in tea leaves from the HM tea plantation suggested that they could have increased contents of human health-related chemicals.

Terpenoids contribute to the floral and fruity aromas of tea^35^. Tea leaves with higher terpenoid levels have an increased flavour level. The expression of terpenoids synthases gene may be stimulated when the plant is under insect attack^36,37^. Higher terpenoid levels in the tea leaves from CK and HM tea plantations, which had higher pest populations than the CP plantation, were hypothesized to result from biostress induced by these pests. Furthermore, in agreement with our speculation, the jasmonate level was lowest in tea leaves from the CP tea plantation (data not shown). Although CP application allowed the tea plantation to maintain a high yield, the flavour of the tea leaves was significantly impacted, which reduced tea quality.

**Figure 8:**
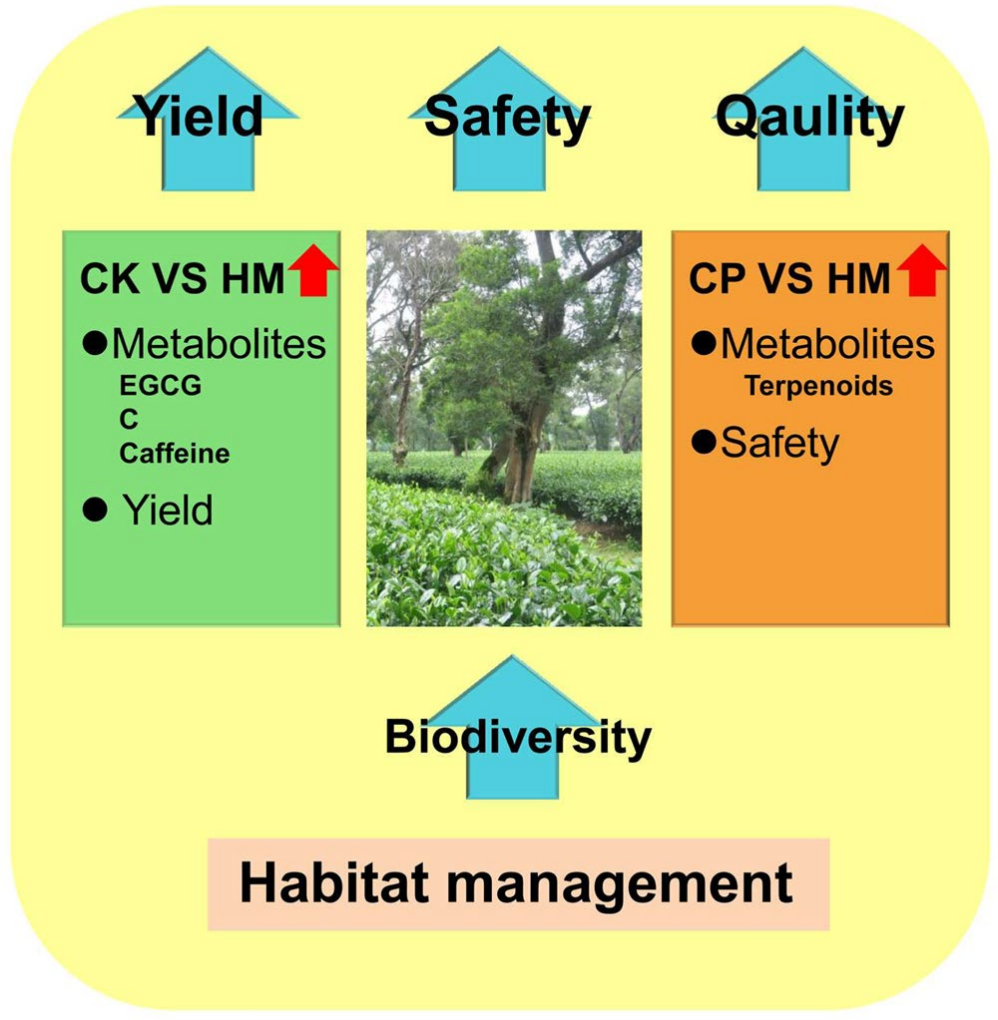
Summary. CK, control; HM, habitat management; CP, chemical pesticide; EGCG, epigallocatechin-3-gallate; C, catechin.

In most crops, habitat management has been applied to safely control pests and maintain yield levels. Limited research has focused on the impact of habitat management on crop quality. Herein, habitat management effectively controlled pests by maintaining arthropod biodiversity and developing natural enemies on the tea plantation. Furthermore, intercropping and natural pest control, as aspects of habitat management, increased the yield. An analysis of metabolites showed that, compared with the CK tea plantation, the levels of some catechins, including EGCG, were significantly increased on the HM tea plantation. Additionally, owing to plant responses to biostress and a larger arthropod community on the HM tea plantation, aroma compounds were more abundant in tea leaves from the HM tea plantation than in those from the CP tea plantation. Therefore, the tea quality appeared to be better on the HM tea plantation than on the CP tea plantation. Accordingly, considering yield, safety, and quality, habitat management was the best approach for operating a tea plantation (Fig. 8). This study demonstrates the dual function of habitat management in pest control and tea quality improvement for the first time, which strongly supports the future wide application of habitat management in tea plantations.

## Methods

### Study area and experimental design

This study was conducted in the city of Yingde (24°18′09″–24°18′15″N, 113°23′09″–113°23′09″) in Guangdong Province, China. The area is located at an altitude of 38–40 m. *Camellia sinensis* (L.) O. Kuntze cv. Yinghong No. 9 was used as the experimental tea variety.

No CPs were applied to the control tea plantation (CK), which lacked a buffer zone and had relatively few surrounding landscape plants.

The tea plantation undergoing habitat management (HM) was intercropped with *Acacia confusa* Merr. trees, which were more than 30 years old and 3–5 m high. The planting specification for row spacing was 8 m × 24 m, with 50 plants per hectare. A variety of evergreen landscape trees, including *Cinnamomum burmannii* (Nees & T. Nees) Blume, *Elaeocarpus sylvestris* (Lour.) Poir., and *Cinnamomum camphora* (L.) Presl., were planted around the tea plantation. These trees were 10 years old and 5–10 m high. A variety of winter deciduous tree species were planted in the centre of the tea plantation, including *Michelia alba* D.C., *Magnolia liliiflora* Desr., and *Ginkgo biloba* L. CPs were not applied in the HM tea plantation.

The CP tea plantation was similar in landscape to the CK tea plantation. However, CPs were used to control pests. CPs were generally applied 2–3 times per month from mid-April to late October. The surrounding landscape plants were also relatively few and there was no buffer zone.

All three tea plantations had the same slope and contained five 15 m × 15 m plots. The minimum distance between plots was 7 m. Each plot was more than 7 m from the edge of the tea plantation to eliminate edge effects. The cultivated tea variety was Yinghong No. 9 using six-year-old soil. The soil type, fertilization schedule, and other tea plantation management methods were the same among plantations. The tea picking period was from April to October, with tea leaves picked every seven days.

### Arthropod community investigation

In December 2016, a random sampling method was used to select 10 survey points. Two points that were 10 meters apart were then randomly selected in each plot at each tea plantation. The survey was conducted a total of 24 times, once in the middle of each month and once in the second half of each month. Each quadrat had a tea line that was 1 m in length. The numbers of arthropod species were determined on two plant branches from the upper, middle, and lower layers. Arthropods in the surface soil of each plantation were also recorded. Each quadrat was 1 m^2^. The orders of the arthropods were identified and classified.

### Tea plantation yield indices

From 2014 to 2016, the yield indices, including the lengths of one bud and two-leaves, hundred-bud weights, germination densities, and yields, were analysed. Five sampling points were used, with mining faces of 33 cm × 33 cm at each point. The germination densities, lengths of buds with two leaves, and hundred-bud weights were recorded. The total yield was calculated based on the germination density and hundred-bud weight.

### Tea pesticide residue assay

Chlorfenapyr and abamectin were determined according to the National Standard of China T1379–2007^38^. Imidacloprid was determined according to the National Standard of China NY/T 1724–2009^39^. β-Cypermethrin was determined according to the National Standard of China NY/T 761–2008^40^. Bifenthrin was determined according to the National Standard of China GB/T 5009.146–2008^41^. Isocarbophos was determined according to the National Standard of China GB/T 5009.109–2003^42^.

### Endogenous volatile analysis

Briefly, finely powdered single bud with two leaves (1 g) was extracted with CH_2_Cl_2_ (6 mL) containing ethyl *n*-decanoate (5 nmol) as an internal standard for 8 h. The mixture was then dried over anhydrous sodium sulfate, and 1 μL of the filtrate was subjected to GC–MS analysis using a SUPELCOWAX 10 column (Supelco Inc., 30 m × 0.25 mm × 0.25 μm). Helium was the carrier gas, with a flow rate of 1.0 mL/min. The initial temperature was maintained at 60 °C for 3 min, then ramped to 240 °C at 4 °C/min and maintained at 240 °C for 20 min. MS analysis was conducted in full scan mode (mass range, *m/z* 40–200).

### RNA extraction, cDNA library preparation, and transcriptome sequencing

Total RNA was extracted from tea leaves (1 g) using the CTAB method^43^. RNA integrity was confirmed using an Agilent 2100 Bioanalyzer with a minimum integrity number of 8. mRNA was purified from total RNA using oligo (dT) magnetic beads and then fragmented. The cDNA library was created using these cleaved RNA fragments and sequenced using the Illumina Hiseq 2000 platform.

### Metabolite extraction and LC–MS/MS analysis

Briefly, finely powdered tea leaves (100 mg) were extracted with 70% methanol (1 mL) containing lidocaine (0.1 μg/mL) overnight at 4 °C. The samples were centrifuged at 10,000 × *g* for 10 min. The supernatant was filtered (0.22 μm) and subjected to UPLC–MS/MS analysis. Analysis was conducted using a Shim-pack UFLC SHIMADZU CBM20A instrument equipped with an ACQUITY UPLC HSS T3 C18 column (Waters, 1.8 μm × 2.1 mm × 100 mm). The injection volume was 5 μL and the column temperature was 40 °C. Gradient elution was conducted using a binary solvent system consisting of 0.04% HOAc in H_2_O (solvent A) and MeCN (0.04% HOAc) (solvent B) at a constant flow rate of 0.4 mL/min. A linear gradient profile with the following proportions (v/v) of solvent B was applied: 0 min, 5%; 0–11 min, 95%; 11–12 min, 95%; and 12–15 min, 5%. In the API 4500 QTRAP LC–MS/MS system (AB SCIEX, CA, USA), the electrospray ionization temperature was 550 °C, the capillary voltage was 5500 V, and the curtain gas pressure was 25 psi. Data were analysed using Analyst 1.6.1 software (AB SCIEX).

### Data statistics and analysis

One-way ANOVA was conducted using IBM SPSS statistics 19. P < 0.05 was considered significant.

## Electronic supplementary material

Supplementary information

## Data Availability

All data generated or analysed during this study are available from the corresponding author on reasonable request.
