# Supplementary material for: Habitat management as a safe and effective approach for improving yield and quality of tea (Camellia sinensis) leaves
Source: Sci Rep. 2019 Jan 23;9:433. doi: 10.1038/s41598-018-36591-x (PMC6344551; doi:10.1038/s41598-018-36591-x)
Supplement: Supplementary file 1 — Supplementary information [file 41598_2018_36591_MOESM1_ESM.docx]

**Title:** Habitat management as a safe and effective approach for improving yield and quality of tea (*Camellia sinensis*) leaves

**Authors:** Jianlong Li ^1, †^, Ying Zhou ^2,3 †^, Bo Zhou ^1^, Hao Tang ^1^, Yiyong Chen ^1^, Xiaoyan Qiao^1^, Jinchi Tang^1*^

***Affiliation*:**

*^1^Tea Research Institute, Guangdong Academy of Agricultural Sciences and Guangdong Provincial Key Laboratory of Tea Plant Resources Innovation and Utilization, Dafeng Road 6, Tianhe District, Guangzhou 510640, China*

*^2^Guangdong Provincial Key Laboratory of Applied Botany & Key Laboratory of South China Agricultural Plant Molecular Analysis and Genetic Improvement, South China Botanical Garden, Chinese Academy of Sciences, Xingke Road 723, Tianhe District, Guangzhou 510650, China*

*^3^University of Chinese Academy of Sciences, No.19A Yuquan Road, Beijing 100049, China*

^*^ Corresponding author. Jinchi Tang, Tel: +86-20-85161049; Email address: tangjinchi@126.com.

^†^ These authors contributed equally to this work.

**Supplementary Information**

**Table** **S1** Analysis on arthropod diversities in different tea plantations in 2016

| Tea types | Diversity index | Evenness index | Richness index | Dominance index |
| --- | --- | --- | --- | --- |
| CK | 0.97±0.02a | 0.71±0.01a | 9.43±0.36a | 0.16±0.01b |
| HM | 1.02±0.03a | 0.73±0.02a | 9.86±0.38a | 0.16±0.02b |
| CP | 0.87±0.03b | 0.72±0.02a | 7.84±0.33b | 0.23±0.02a |

Data are expressed as mean ± S. D. (n=24). Different letters indicate significant differences (p < 0.05 ).


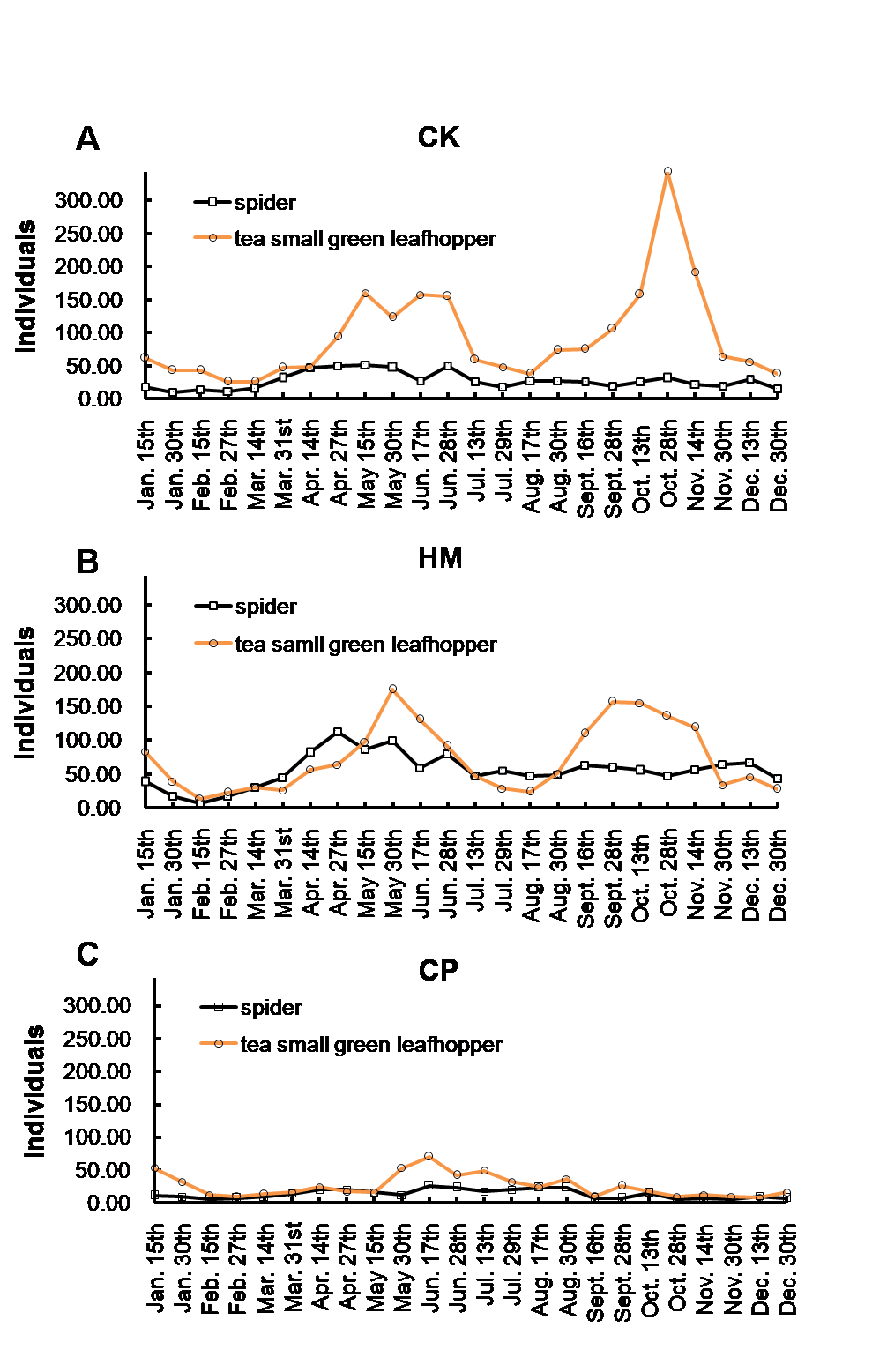


**Figure S1.** The trends of spider and tea small green leafhopper in CK, HM and CP tea plantation in 2016. The x-axis represents the day the insects recorded.


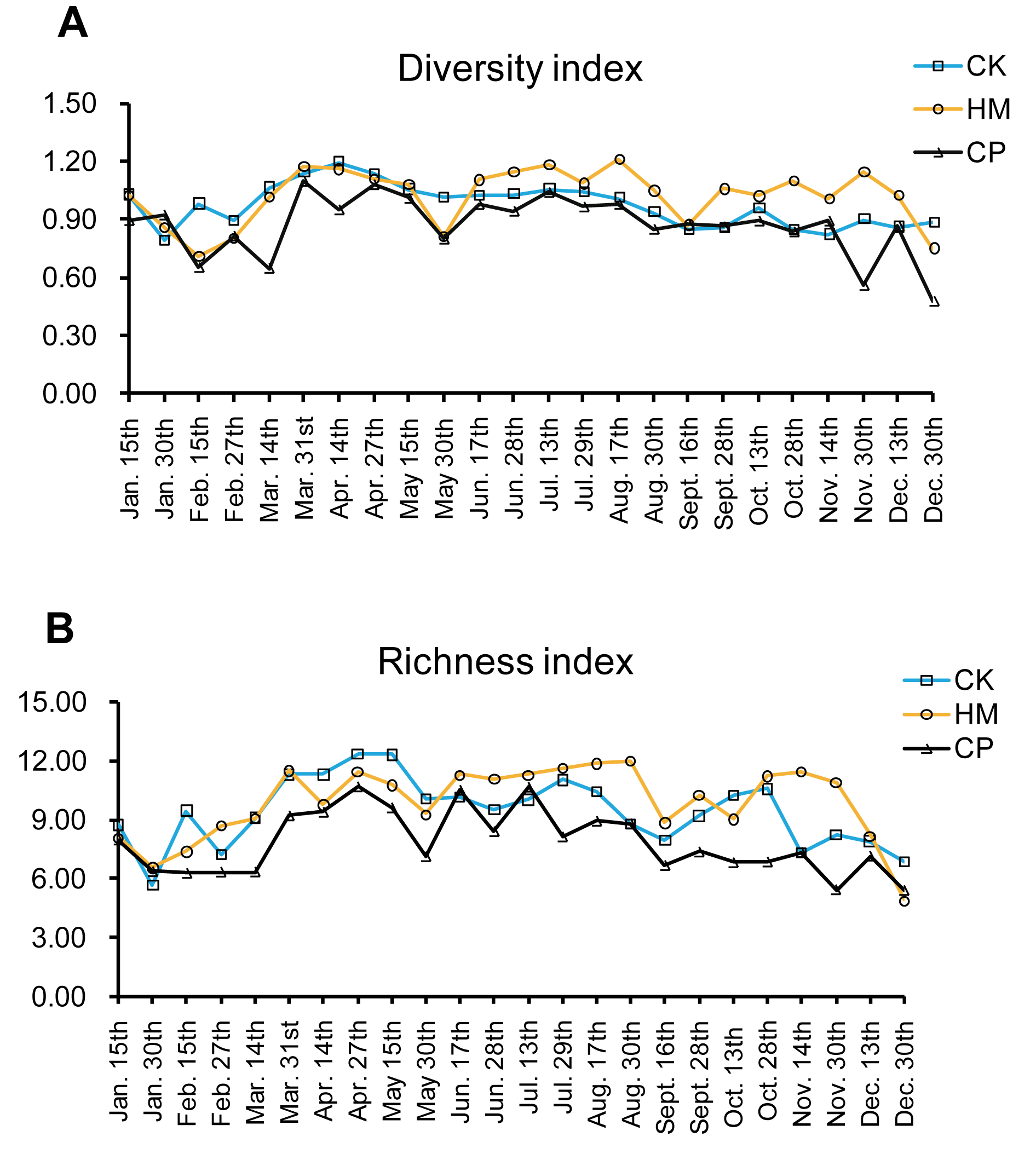


**Figure S2.** The trends of diversity index and richness index in 2016. The x-axis represents the day the insects recorded.
